# Heart rate response to cognitive load as a marker of depression and increased anxiety

**DOI:** 10.3389/fpsyt.2024.1355846

**Published:** 2024-07-11

**Authors:** Evgeniia I. Alshanskaia, Natalia A. Zhozhikashvili, Irina S. Polikanova, Olga V. Martynova

**Affiliations:** ^1^ School of Psychology, Faculty of Social Sciences, HSE University, Moscow, Russia; ^2^ Faculty of Social Sciences, Laboratory for Cognitive Research, HSE University, Moscow, Russia; ^3^ Faculty of Biology and Biotechnology, HSE University, Moscow, Russia; ^4^ Institute for Cognitive Neuroscience, HSE University, Moscow, Russia; ^5^ Laboratory of Human Higher Nervous Activity, Institute of Higher Nervous Activity and Neurophysiology of the Russian Academy of Sciences, Moscow, Russia

**Keywords:** cognitive stress, electrocardiogram, photoplethysmogram, heart rate, HRV, depression, anxiety

## Abstract

**Introduction:**

Understanding the interplay between cardiovascular parameters, cognitive stress induced by increasing load, and mental well-being is vital for the development of integrated health strategies today. By monitoring physiological signals like electrocardiogram (ECG) and photoplethysmogram (PPG) in real time, researchers can discover how cognitive tasks influence both cardiovascular and mental health. Cardiac biomarkers resulting from cognitive strain act as indicators of autonomic nervous system function, potentially reflecting conditions related to heart and mental health, including depression and anxiety. The purpose of this study is to investigate how cognitive load affects ECG and PPG measurements and whether these can signal early cardiovascular changes during depression and anxiety disorders.

**Methods:**

Ninety participants aged 18 to 45 years, ranging from symptom-free individuals to those with diverse psychological conditions, were assessed using psychological questionnaires and anamnesis. ECG and PPG monitoring were conducted as volunteers engaged in a cognitive 1-back task consisting of two separate blocks, each with six progressively challenging levels. The participants’ responses were analyzed to correlate physiological and psychological data with cognitive stressors and outcomes.

**Results:**

The study confirmed a notable interdependence between anxiety and depression, and cardiovascular responses. Task accuracy decreased with increased task difficulty. A strong relationship between PPG-measured heart rate and markers of depression and trait anxiety was observed. Increasing task difficulty corresponded to an increase in heart rate, linked with elevated levels of depression and trait anxiety. A strong relationship between ECG-measured heart rate and anxiety attacks was observed. Increasing task difficulty corresponded to an increase in heart rate, linked with elevated levels of anxiety attacks, although this association decreased under more challenging conditions.

**Discussion:**

The findings underscore the predictive importance of ECG and PPG heart rate parameters in mental health assessment, particularly depression and anxiety under cognitive stress induced by increasing load. We discuss mechanisms of sympathetic activation explaining these differences. Our research outcomes have implications for clinical assessments and wearable device algorithms for more precise, personalized mental health diagnostics.

## 1 Introduction

The escalating global health burden of cardiovascular and mental health disorders, highlighted by the World Health Organization (1), indicates the need for advanced preventative strategies and interventions. Cognitive stress, also known as mental stress (2, 3), refers to the ways in which stress factors such as increased cognitive load affect mental processes. Cognitive stress is a crucial factor that links physiological and psychological well-being to mental health conditions such as depression and anxiety. It affects cognition and emotional processing (3, 4). The complex interplay between heart activity, stress, and cognitive function has been examined (5, 6) establishing connections between cognitive decline, vascular health, and mental well-being (7). Heart rate variability (HRV) has emerged as a focal point, particularly in its correlation with depression and anxiety (6, 8–12). Studies have consistently shown that HRV, which reflects the autonomic nervous system’s ability to respond to various psychological and physiological stimuli, is closely linked with mental health conditions and cognitive load (5, 13). Additionally, heart rate mean (HR) as a biomarker for mental disorders has been determined (13–15).

Application of photoplethysmography (PPG), a non-invasive method for measuring blood volume changes in the microvascular bed of tissue, for assessing heart rate offers a different lens through which to view the heart’s response to stress. Recent studies highlight the importance of peripheral vasoconstriction, governed by the sympathetic nervous system, as an indicator of mental effort and stress levels (16–19). There is a significant physiological connection between PPG amplitude variations and stress responses, as well as cognitive load (20–22). PPG is also used in the context of anxiety and depression assessment (23). This measurement provides critical insights into the neurophysiological mechanisms underpinning stress, anxiety, and depression (24, 25).

Electrocardiogram (ECG) provides cardiac indicators that enhance our understanding of stress-related physiological responses. ECG monitors heart rate and contractility, predominantly influenced by adrenergic receptors response (26, 27). This cardiac response is reflected in the vascular activity, measurable through photoplethysmography. PPG measures peripheral vascular tone, primarily affected by noradrenergic receptors (28), as well as adrenergic receptors (29, 30). These differences could provide critical insights into the neurophysiological mechanisms underpinning stress, anxiety, and depression (24, 25). Understanding mental health disorders can be aided by concurrently examining cardiac and vascular responses (31). The integration of ECG with PPG is advancing in AI-driven personalized diagnostics and wearable technologies (32–34).

Our study addresses the reactivity of ECG and PPG to stress by examining physiological and cognitive responses to modulated cognitive load in a larger sample size of participants with a range of mental conditions. Psychological assessments were conducted for all participants. In the pilot study, we observed a gradual increase in the sympathetic activity of the autonomic nervous system, correlating with respiratory and cardiovascular responses under cognitive load (35) and linking patterns of the autonomic nervous system (ANS) with questionnaire responses (36).We hypothesized that ECG and PPG HRV parameters would demonstrate the most pronounced association with traits of depression and anxiety, predicated on the assumption that the complexity level of tasks. This enhances our understanding of the mechanisms underlying anxiety and depression.

## 2 Materials and methods

### 2.1 Participants

A cohort of 95 individuals, initially recruited between the ages of 19 and 48 years (average age = 25 ± 7.8; 61 females), participated in the study. After removing individuals below 18 or above 45 years due to technical issues in their data recordings, 90 subjects (average age = 25 ± 7.6; 57 females) were included in the final analysis. Participants exhibiting traits of mental disorders were recruited through referrals from practitioners, while individuals with normal mental traits were invited via social networks and the faculty’s public website. Uniform assessments, consisting of questionnaires and medical histories, were conducted for all participants following a standardized protocol that ensured confidentiality and data anonymization. Participants were classified based on questionnaire responses, self-reported data, and preliminary diagnoses, all evaluated by an expert using normative parameters, facilitating a precise distinction between normative and non-normative traits.

### 2.2 Questionnaires and psychological assessment

The participants underwent a detailed anamnestic review to report their experiences with anxiety attacks and depressive episodes, using the following encoding systems. Anxiety attacks were assessed using a binary scale, where ‘0’ indicated no anxiety attacks during the individual’s lifespan and ‘1’ indicated the presence of any anxiety attacks at any point in the past five years. This scale was chosen due to the self-reported nature of the information and the necessity of a two-parameter approach for mental health professionals. Episodes of depression were evaluated on a three-point scale. A score of ‘0’ was given if there were no episodes of depression throughout the individual’s life. A ‘0.5’ was assigned if episodes of depression were self-reported without the involvement of mental health professionals. Likewise, a score of ‘1’ was used when episodes of depression were both self-reported and confirmed by a mental health practitioner. This approach allowed for precise differentiation between self-reported anxiety and various levels of depression. Key psychological assessments included the Beck Depression Inventory (BDI-II) (37–40) and the State-Trait Anxiety Inventory, Trait Version (STAI-T) by Spielberger (41, 42), to quantify depressive symptoms and differentiate between state and trait anxiety. Our study analyzes self-reported questionnaire data to comprehensively analyze traits of anxiety and depression. We included both participants referred by mental health practitioners and those recruited independently, assessing their mental states using validated inventories. Employing a triangulation approach, we aimed to differentiate symptoms in our assessment. In subsequent text, we will mention traits of depression (BDI), traits of anxiety (STAI), as well as self-reported depressive episodes and self-reported anxious episodes. If both depression-related evaluations (BDI-II and self-reported episodes) align, we will label it as ‘depression’. Similarly, if anxiety-related evaluations (STAI-T and self-reported episodes) align, we will label it as ‘anxiety’.

### 2.3 Stimuli and procedure

For inducing cognitive stress, we employed the Cognitive 1-back Colour Matching Task (CMT) (43). This approach was selected to balance the challenge across participants while avoiding cultural and educational bias, making it particularly suitable for diverse study populations. This task was tested in preliminary studies, which confirmed its effectiveness at inducing cognitive load without exceeding participant capabilities. Furthermore, the level of task difficulty can be adjusted by increasing the number of objects that participants must remember, transitioning to a 2-back or 3-back design. In our study, we regulated difficulty by altering the object count. This approach ensures a controlled increment in cognitive load, facilitating a more precise evaluation of cognitive stress induced by memory tasks as tested in previous research (35, 44).

**Figure 1:**
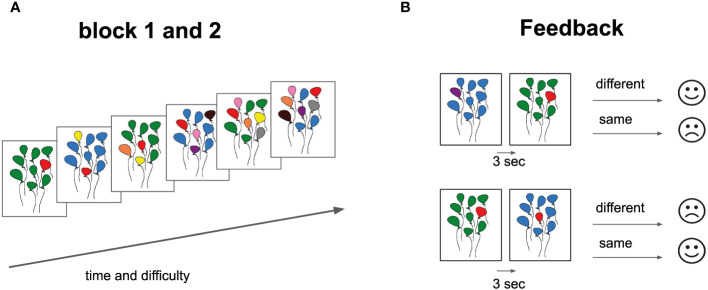
Cognitive Task Design and Feedback Structure. **(A)** Task Progression: Each block displays six balloon bundles, representing a gradient in difficulty from left (simpler configurations) to right (complex configurations). **(B)** Feedback Component: Two paired balloon bundle sets are presented. Adjacent feedback icons (straight-mouth for “same” and curved-mouth for “different”) provide responses post a 3-second interval. This design assesses participants’ ability to discern and recall balloon color patterns, with immediate feedback facilitating performance tracking.

Participants were instructed to view images of colored balls and compare each with the preceding one to identify color matches. Key 1 was designated for matches, and key 2 for mismatches, with a primary emphasis on color rather than position. The task consists of two parts, each comprising six escalating difficulty levels. Each level includes 17 trials, totaling 102 samples per block (204 samples for 2 blocks). Among these, 49 trials required a ‘2’ response for non-matching samples, and 41 necessitated a ‘1’ response for matching samples. It commences with a simple task of recognizing a single-color change in an image with balloons and progressively escalates to identifying up to six color changes. Each image was displayed for one second, with a 30-second rest provided after each block. Task completion for each level took about a minute, and a block took approximately seven minutes, contingent on participants’ response speed. The entire experiment lasted no more than 15 minutes per participant, with limited time for answering. Participants received visual feedback after each trial (**Figure 1**). Immediate feedback was provided after each response, indicating correctness, incorrectness, or responses exceeding three seconds. After completing each level, participants evaluated their performance.

We assessed how participants managed cognitive stress by correlating their physiological responses with their accuracy in providing correct answers. Participants’ rewards included a base amount for participation, supplemented by a performance-based bonus calculated according to the number of correct answers, following the methodology employed in the pilot study (35). The decision time (the time from the appearance of the second image with balloons to pressing the key to answer) for each block and level was analyzed separately. Stimuli sequence and data for the analysis of behavioral responses were obtained using Eye-Link Experiment Builder 2.3.1 (Mississauga, Ontario, Canada: SR Research Ltd., 2020) synchronized with ECG and PPG recording.

### 2.4 Recording and signal processing

ECG and PPG data were continuously recorded to assess autonomic nervous system reactions using a rheograph-polyanalyzer RGPA-6/12, with a sampling rate of 250 Hz and a filtration range of 0.5–75 Hz. ECG measurements utilized sensors strategically placed on the right and left wrists, as well as the right ankle. Simultaneously, PPG data registration employed sensors positioned on the distal phalanx of the middle finger on the left hand. The initial data processing was conducted using Neurokit2 (45), specialized software designed for such tasks. It featured a custom processing function tailored specifically for ECG and PPG signal types, ensuring comprehensive signal purification and subsequent peak detection.

For the ECG dataset, a fifth-order Butterworth high-pass filter with a cutoff frequency of 0.5 Hz was applied, in combination with a 50 Hz power line filter. The QRS complexes in the ECG signal were identified based on their gradient steepness, with R-peaks recognized as local maxima (45, 46). When processing Photoplethysmography (PPG) data, a third-order Butterworth filter with a passband ranging from 0.5 to 8 Hertz was employed. The processing methodology described in Method IV (47) was applied to PPG signals for data purification and peak detection. This approach, used for detecting systolic peaks in acceleration photoplethysmograms (APG) signals, involves a three-stage algorithm: pre-processing with bandpass filtering and squaring the signal, feature extraction using moving averages to identify potential systolic peaks, and classification through dynamic thresholding for accurate peak determination. This method, integrated into the NeuroKit2 toolkit, effectively removed extraneous noise and artifacts from the PPG signal, ensuring cleaner and more reliable data for analysis.

Mean cardiac parameters, including HRV time and frequency domains, were calculated for each block level from both ECG and PPG signals, including Heart Rate, High Frequency (HF), Low Frequency (LF), MeanNN (the average of all normal-to-normal intervals), Root Mean Square of Successive Differences (RMSSD), and Standard Deviation of NN intervals (SDNN), thereby providing a comprehensive analysis of autonomic nervous system activity. Following the initial processing with Neurokit2, an issue of over-detection of R-peaks in the ECG dataset became apparent, suggesting the presence of extraneous signals and peaks. The BioPsyKit toolkit, with artifact detection capabilities (48), was utilized to filter out extraneous peaks employing the rooted algorithm (49).

### 2.5 Statistical analysis

To test the hypotheses, we first computed the mean heart rate measures and response accuracy for each individual at different task difficulty levels. Subsequently, we employed the task difficulty level, ranging from 1 to 6 colors, as a categorical variable in our analysis. This approach was crucial to ensure that we did not overlook potential non-linear relationships and interactions with task difficulty.

#### 2.5.1 Association of heart rate indicators with depression and anxiety

We set out to determine which HRV indicator has the strongest association with anxiety and depression. For each heart rate variable and each anxiety/depression variable, we applied a linear mixed effects model using the nlme package in R (50). The model was specified as follows: heart rate ~ anxiety/depression * difficulty + (~1 | ID), where the dependent variable was heart rate, and the fixed effects included the anxiety/depression score and task difficulty. We included a random intercept for participant code (ID). The random intercept was consistently utilized throughout this analysis to account for individual differences in the target variable, reducing the impact of individual variability. Model comparison was conducted using the Akaike Information Criterion (AIC), a metric for assessing information loss in a model (51). To ensure fair comparisons via AIC, we standardized the target variables to a common scale using the z-scaling method.

#### 2.5.2 Relationship between depression, anxiety, and heart rate response to task difficulty

The next aim was to determine how HRV is related to task difficulty, anxiety, and depression. Subsequently, for each depression (BDI depression, self-reported episodes of depression) and anxiety (STAI trait anxiety, self-reported anxiety attacks) measure, we examined the 4 models that exhibited the best performance according to AIC. To assess the model coefficients, we employed Wald t-statistics with a significance threshold of p <.01. Additionally, these models were replicated with the addition of gender as a predictor (see **Supplementary Materials** for results).

#### 2.5.3 Relationship between heart rate response to task difficulty and task-solving accuracy

Furthermore, for heart rate indicators displaying the most robust relationships with depression and anxiety, we developed linear mixed effects models with the following formulation: heart rate ~ accuracy * difficulty + (~1 | ID). We evaluated the coefficients of these models using Wald t-statistics, maintaining a significance threshold of p < 0.01.

## 3 Results

### 3.1 Basic behavioral statistics

The Pearson correlation test revealed a significant association between trait anxiety (STAI; mean = 44.7 ± 8.9) and trait depression (BDI; mean = 9.3 ± 7.1; r = 0.6, p < 0.001). Two-way ANOVA tests indicated that significant relationships between depression episodes and anxiety attacks with STAI trait anxiety (F (2, 84) = 3.95, p = 0.02; F (1, 84) = 11.84, p < 0.001; respectively), as well as with BDI depression (F (2, 84) = 19.04, p < 0.001; F (1, 84) = 27.38, p < 0.001; respectively). However, depression episodes were not significantly associated with anxiety attacks (χ² = 5.21, p = 0.07). Accuracy, measured as the percentage of correct answers (86.7% ± 5.1%), decreased as task difficulty increased (r = -0.75, p < 0.001). Accuracy was not related to anxiety (r = -0.06, p = 0.56), depression (r = 0.06, p = 0.56), depression episodes and anxiety attacks (F = 0.23, p = 0.79; F = 0.15, p = 0.7; relatively). Descriptive statistics for response accuracy, HRV and psychological indicators can be found in **Table 1**.

**Table 1:** Analysis results, delineating physiological and psychological variable values categorized by groups.

	Self-reported depression			Self-reported anxiety attacks	
	None	Self-diagnosed	Psychiatrist-diagnosed	None	Self-reported
Accuracy (%)	86.7	88	86.7	86.7	87.3
STAI-T	42.9	42.9	50.1	42.6	50.3
BDI	6.4	11.2	15.0	7.0	15.7
PPG HF	0.07	0.06	0.07	0.07	0.07
PPG LF	0.03	0.03	0.03	0.03	0.03
PPG MeanNN	780.6	736.1	773.9	782.6	738.9
PPG RMSSD	102.6	109.7	106.5	107.1	98.1
PPG SDNN	83.6	88.7	83.4	87.2	76.6
PPG Rate mean	79.2	83.8	79.5	78.9	83.3
ECG HF	0.04	0.04	0.04	0.04	0.04
ECG LF	0.03	0.03	0.03	0.03	0.03
ECG MeanNN	783	746	779	789.5	736.5
ECG RMSSD	48.3	41.1	34.7	43.7	43.8
ECG SDNN	55.0	48.6	43.8	51.4	50.2
ECG Rate mean	78.9	82.9	78.8	78.3	83.3
PPG Amplitude	0.506	0.47	0.558	0.498	0.55

### 3.2 Heart rate variability indicators and depression and anxiety traits

Initially, we identified the HRV indicator most strongly associated with anxiety and depression (refer to Subsection 2.5.1 in Methods). Models incorporating trait anxiety (STAI), trait depression (BDI), and episodes of depression as predictors indicated that the PPG mean heart rate had the lowest information loss (AIC = 134, AIC = 133.7, AIC = 132.3, respectively). For models involving anxiety attacks, the ECG mean heart rate model showed the lowest information loss (AIC = 141). AICs of all models can be found in **Table 2**. Thus, the analysis revealed a statistically significant association between mean heart rate and depression and anxiety indicators, which was more pronounced than with other HRV parameters. Furthermore, the examination of PPG-derived mean heart rate data indicated significant statistical associations with parameters such as personal anxiety, self-reported depression, and diagnosed depression. Concurrently, ECG-measured mean heart rate dynamics demonstrated a more pronounced association with episodes of anxiety attacks.

**Table 2:** Akaike Information Criterion (AIC) of 48 models predicting heart rate parameters through anxiety/depression and task difficulty.

		Depression (BDI)	Anxiety (STAI-T)	Depression episodes (self-reported)	Anxiety attacks (self-reported)
ECG	HF	1133.407	1133.581	1144.643	1134.657
ECG	LF	1166.415	1165.558	1168.916	1166.676
ECG	MeanNN	141.5409	138.6218	145.5449	141.3978
ECG	RMSSD	1060.103	1052.215	1067.73	1061.822
ECG	SDNN	1155.019	1143.481	1167.177	1161.547
ECG	Rate mean	143.8256	143.3636	145.4342	**140.9845**
PPG	HF	1107.02	1105.825	1110.521	1108.845
PPG	LF	1124.783	1122.753	1116.137	1122.649
PPG	MeanNN	154.8627	151.677	152.8998	156.3769
PPG	RMSSD	782.9634	772.0694	784.9265	784.7503
PPG	SDNN	879.5336	870.5809	884.8129	882.2574
PPG	Rate mean	**133.6984**	**133.9978**	**132.3324**	142.9038
PPG	Amplitude	578.4	575.3	579.9	576.6

The best (lowest) AIC scores for each anxiety/depression measure are shown in bold.

### 3.3 Mean heart rate, task difficulty and depression and anxiety traits

Subsequently, we explored how selected HRV indicators correlate with task difficulty, anxiety, and depression (refer to Subsection 2.5.2 in Methods). For the examination of trait anxiety (STAI), trait depression (BDI), and self-reported depression episodes, we analyzed models predicting PPG heart rate. For the analysis of self-reported anxiety attacks, we utilized models predicting ECG heart rate mean (as discussed in Section 3.2).

**Figure 2:**
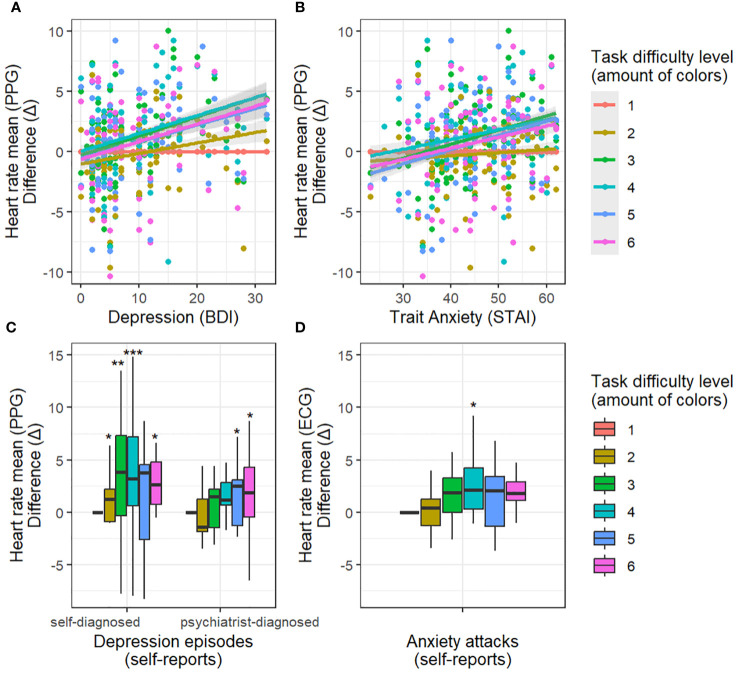
Dependency between depression/anxiety and heart rate with increasing task difficulty relative to the easiest task condition. Difference (Δ) - difference in heart rate between the easiest difficulty level (1 color) and other difficulty levels. Top panels: results for depression **(A)** and anxiety **(B)** assessed with questionnaires (BDI, STAI-T). The more severe the depression/anxiety scores, the greater the increase in heart rate during difficult conditions. Low panels: results for self-reported depression **(C)** and anxiety **(D)** episodes in relation to healthy subjects. Self-reported depression episodes (self-diagnosed, psychiatrist-diagnosed) enhance the effect of heart rate increase during difficult conditions. Self-reported anxiety episodes enhance the effect of heart rate increase during the medium difficulty condition (4 colors). * p-value < 0.05, ** p-value < 0.01, *** p-value < 0.001

The mean heart rate demonstrated a positive association with trait anxiety (STAI) in the challenging task levels (3, 4, 5, 6 colors of balloons) when compared to the easiest task level (interaction between task difficulty and trait anxiety (STAI): t = 3.09, p < 0.01; t = 2.16, p = 0.03; t = 3.13, p < 0.01; t = 2.44, p = 0.02; respectively; refer to **Figure 2**). Additionally, the analyzed model unveiled a main effect indicating a negative association between task difficulty (3 and 5 colors) and the mean heart rate when contrasted with the easiest level (t = -2.36, p = 0.02; t = -2.66, p = 0.008; respectively). The mean heart rate also exhibited a positive association with episodes of anxiety attacks in the middle difficulty task level (4 colors of balloons) when compared to the easiest task level (interaction between task difficulty and anxiety attacks: t = 2.61, p < 0.01) (**Figure 2**).

Furthermore, the mean heart rate displayed a positive association with BDI ratings of depression in the demanding task levels (3, 4, 5, 6 colors of balloons) relative to the easiest task level (interaction between task difficulty and trait depression: t = 3.61, p < 0.001; t = 3.21, p < 0.01; t = 3.03, p < 0.01; t = 3.36, p < 0.001; respectively) as illustrated in **Figure 2**.

Additionally, the mean heart rate exhibited a positive relationship with self-reported episodes of depression in the challenging task conditions (2, 3, 4, 6 colors of balloons) in comparison to the easiest condition (t = 2.45, p = 0.02; t = 3.27, p < 0.01; t = 3.44, p < 0.001; t = 2.31, p = 0.02; respectively; **Figure 2**). A similar effect was observed with self-reports of a formal psychiatrist diagnosis of depression (5 colors: t = 2.02, p = 0.04; 6 colors: t = 2.08, p = 0.04).

### 3.4 *Post-hoc* analysis: heart rate, task difficulty and task accuracy

The analysis unveiled a connection between the mean heart rate and accuracy. Specifically, the mean ECG heart rate exhibited a positive association with accuracy (main effect: t = 3.23, p = 0.001), although this effect diminished in the most challenging condition involving 6 colors (interaction between task difficulty and accuracy: t = -2.03, p = 0.04). Additionally, the analyzed model indicated a main effect of a positive relationship between task difficulty (4 and 5 colors of balloons) and the mean heart rate, in comparison to the easiest level (t = 2.25, p = 0.03; t = 2.5, p = 0.01; respectively).

**Figure 3:**
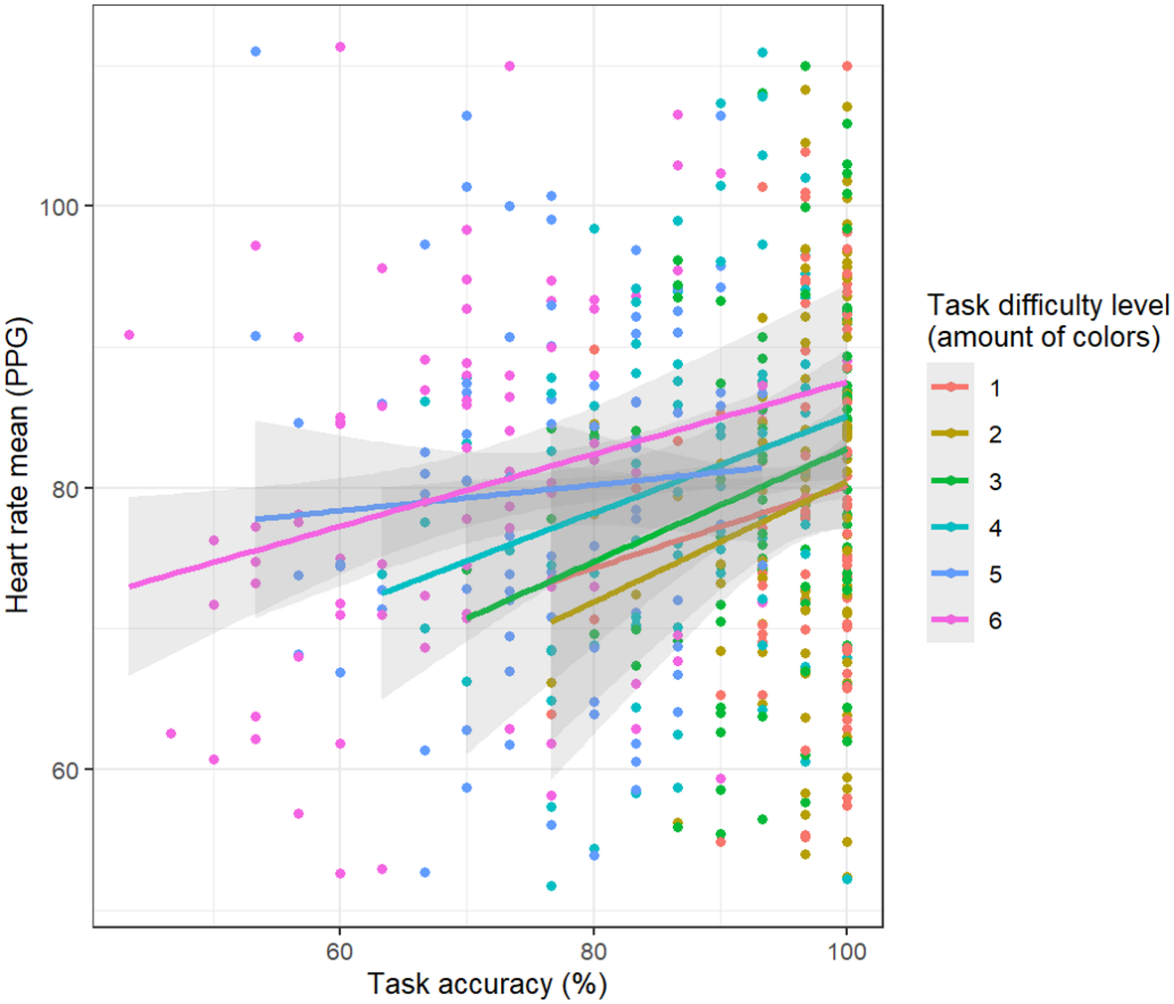
Relationship between task-solving accuracy and mean heart hate derived from PPG data. The higher the heart rate, the greater the accuracy.

Similarly, PPG heart rate displayed a positive association with task-solving accuracy (t = 4, p < 0.001) (**Figure 3**). However, this relationship significantly diminished in more challenging conditions (interaction between task difficulty and accuracy: t = -2.34, p = 0.01 (3 colors difficulty); t = -2.85, p < 0.01 (4 colors of balloons); t = -2.64, p < 0.01 (5 colors of balloons); t = -2.7, p < 0.01 (6 colors of balloons). The analyzed model also revealed a main effect indicating a positive relationship between task difficulty (3, 4, 5, 6 colors of balloons) and PPG heart rate, when compared to the easiest level (t = 2.57, p = 0.01; t = 3.21, p < 0.01; t = 3.07, p < 0.01; t = 3.21, p < 0.01; respectively).

## 4 Discussion

The key findings revealed a pronounced association between the average heart rate, as measured through photoplethysmography (PPG), and indicators of depression and trait anxiety, more significant than the associations observed with heart rate variability (HRV) parameters derived from electrocardiography (ECG). Furthermore, the data demonstrated that mean heart rate was substantially influenced by task complexity among individuals presenting a spectrum of mental health conditions. This association was particularly pronounced in relation to trait anxiety (STAI) and trait depression (BDI), as well as self-reported depression episodes at elevated levels of task difficulty. It is also crucial to acknowledge that filtering ECG noise can be challenging, which may impact the limitations and results of both ECG and PPG signal preprocessing methods.

It is important to consider what the mean heart rate is measured through the detection of R waves, while PPG-obtained mean heart rate measurement relies on identifying the maxima of the pulse wave. Despite both methods targeting the same physiological indicator, they differ in their signal filtering processes. This distinction might elucidate the observed variance in ‘sensitivity’ within our models. The results demonstrate a substantial positive association between heart rate responses and depression, as well as trait anxiety, markers during challenging tasks, signifying a distinct pattern in individuals with anxiety attacks. Furthermore, *post hoc* analyses suggested a diminishing positive relation between mean heart rate and task accuracy in more complex tasks.

### 4.1 PPG heart rate mean and ECG heart rate mean and depression and anxiety traits

In examining the relationship between heart rate metrics and mental health conditions our research contributes new data to existing discussions. While the focus has traditionally been on HRV (52, 53) as a marker of the parasympathetic system’s adaptability (54, 55), our findings reveal a stronger predictive relationship with the mean heart rate (HR). Distinct from prevalent methods, our study measured HR and HRV during task performance, particularly under conditions of high workload. This contrasts with most existing research on HRV and its relationship to anxiety and depression, which predominantly focuses on resting or recovery phases, omitting the context of task execution (15). Our data suggest that HR stress response in the sympathetic system is more sensitive to conditions of anxiety and depression during challenging tasks than HRV indicators.

Furthermore, it was found that for anxiety and depression as measured by questionnaires, and for self-reported depression episodes, the strongest relation was observed with HR measured by PPG. Thus, despite ECG being considered a more reliable method for measuring HRV (56), and the relative scarcity of research into PPG signals in relation to depression and anxiety, our results suggest that PPG is an effective tool for gauging depression and trait anxiety through HR measurement. Concurrently, the highest connection for self-reported anxiety episodes (anxiety attacks) was observed with HR measured by ECG.

The study highlighted that PPG, a marker for cardiac and blood vessel activity reflecting peripheral vascular tone influenced by noradrenergic receptors, is closely linked with mental health issues, emphasizing the possible significance of noradrenaline’s role in sympathetic activation related to the investigated states and traits. These outcomes are aligned with previous studies (18, 24, 25, 29, 57, 58).The distinct cardiovascular receptor activities in the heart and vessels might explain the differential sensitivity of ECG and PPG in measuring anxiety and depression, despite the heart’s beating wave spreading from the heart to the vessels. β1-Adrenergic receptors, predominantly adrenergic (30, 59) in the heart, which increase heart rate and contractility in stress responses (60), are aligned with ECG measurements during self-reported anxiety attacks. However, PPG’s sensitivity to changes in peripheral blood flow is influenced by α1 receptors, predominantly noradrenergic (61, 62), which are responsible for vasoconstriction and thus their influence is a crucial factor in PPG measurements. These sympathetic vascular changes have a higher association in our study with anxiety and depression symptoms as assessed by questionnaires and self-reported depression episodes. Despite the presence of other factors in vessels, such as β2 adrenoreceptors, NO (nitric oxide), EDRF (endothelial derived relaxing factor), and others (63, 64), and vagal sympathetic regulation M2 in heart (65), these receptor mechanisms dominate their study, in connection with physiological research that can provide additional insights about ANS and CNS in response to stress.

### 4.2 Mean heart rate, task difficulty and depression and anxiety traits

It was found that mean heart rate responses to 1-back working memory task difficulty are related to levels of anxiety and depression, supporting our research hypothesis. Individuals with high depression scores on BDI and high trait anxiety as assessed by the STAI, as well as those with self-reported depression episodes (both self-diagnosed and psychiatrist-diagnosed), exhibited increased HR during challenging task levels. Additionally, the increased mean heart rate was observed during moderately difficult tasks in individuals with self-reported anxiety attacks. However, results for anxiety attacks were not replicated when gender was added to the model (refer to **Supplementary Materials**).

In previous studies, heart rate (HR) responses to stress, depression, and panic attacks have been identified as key areas of investigation (14, 66). Furthermore, it is well-established that tasks demanding executive functioning, which were employed in our study to induce psychological strain, correlate with heightened cardiac indicators, corroborating findings from prior research (31, 67). Within our study, we observed distinct patterns of HR fluctuations associated with cognitive load in individuals experiencing anxiety and depression. It may be inferred that heightened anxiety and depression (both clinical and non-clinical) are linked to an intensified stress response during the execution of difficult working memory tasks. However, contrasting views exist, suggesting that depression is associated with a reduced HR response in depressed individuals to stressful situations (13, 15). Therefore, HR increases in our study might reflect not merely a reaction to stress recovery, but an adaptive response to increasing task difficulty (68). This perspective is supported by studies indicating heightened nervous activity during working memory tasks, particularly among individuals with anxiety traits (44, 69–72).

In our research Trait anxiety (STAI) and trait depression (BDI), along with self-reported episodes of depression, appear to similarly influence the HR response to task difficulty: an increase in HR response is observed in both depression and trait anxiety (**Figure 2**). This observation suggests that our study paradigm was more likely to detect a common physiological effect for trait anxiety (STAI), trait depression (BDI), and self-reported episodes of depression without effectively distinguishing between them. In contrast, only self-reported anxiety attacks demonstrated a notably different effect. HR response to task difficulties was heightened in subjects with reported anxiety attacks, but this was specifically evident at the average level of difficulty (**Figure 2**). In addition, the results showed that, unlike other psychological characteristics, only anxiety attacks showed a better association with ECG (Section 4.2). It is possible that anxiety attacks are associated with distinct β-adrenergic dysregulation (73, 74) and psychophysiological mechanisms, compared to those seen with trait anxiety (STAI), trait depression (BDI), and self-reported episodes of depression. This could be a relevant theme for future research. However, it should be noted that we did not statistically compare the direct effects of depression and anxiety, but rather how they influence cardiac responses.

### 4.3 Heart rate response patterns and task accuracy

In the research, we observed a positive relationship between HR and accuracy across various levels of task difficulty, with a more pronounced correlation in simpler tasks. This suggests that HR may reflect an adaptive stress response, where increased mental effort leads to improved performance, particularly in less complex tasks. However, this finding partly contrasts with previous studies. HR could be indicative of high cognitive load (55), especially in stressful conditions outside of the laboratory. Similarly, Solhjoo (13) observed that while increased cognitive load positively correlated with HRV, mean HR and single-item cognitive load measures exhibited a negative correlation with clinical reasoning performance. This highlights the potential of physiological monitoring as a tool for identifying individuals experiencing high cognitive load and at risk of underperforming in complex reasoning tasks, both with and without mental issues (15). We suggest that the heart rate monitoring could impact diverse perspectives in mental health assessment by integrating biochemical correlations (59), advanced algorithms, personalized Artificial Intelligence approaches with behavioral data, and cardiac biomarkers to provide reliable tools for mental health practitioners.

### 4.4 Limitations

This study, while offering insights and potential for future research, is constrained by its sample size. The variability in individual physiological and psychological responses can significantly impact HR patterns, underlining the need for larger and more diverse participant groups in subsequent studies. While this approach strengthened the clarity of our findings within an adult cohort, it necessitates further research across broader age ranges to ensure the universality and applicability of the results. Another limitation is the reliance on self-reported data, which necessitates more detailed research and refinement. Collaborations with experts and the integration of advanced technologies like artificial intelligence and machine learning could enhance data accuracy and interpretation. Additionally, methodological challenges in handling the noise typical of PPG signals are noteworthy. Preprocessing these signals, while crucial for data clarity, may inadvertently result in the loss of nuanced physiological information. Given known gender differences in the prevalence of anxiety and depression, our results might reflect indirect gender effects rather than direct correlations with anxiety and depression levels. Although we recalibrated our models to include gender as a predictor (refer to **Supplementary Materials**), confirming the significance of certain interactions irrespective of gender, the potential confounding impact of gender requires further investigation (75, 76).

## 5 Conclusion

In summary, this study explores the relationship between heart rate metrics and mental health, specifically focusing on depression and anxiety traits under cognitive stress. Our findings enhance the understanding of physiological markers, emphasizing the importance of HR in addition to HRV, and comparing measurement techniques such as PPG and ECG. The research outcomes underscore the superior predictive capability of heart rate, particularly metrics derived from PPG, in association with depression and anxiety during cognitive tasks. These findings advance our understanding of heart rate metrics as dependable indicators in mental health evaluations under cognitive stress. Heart rate metrics enhance diagnostic precision, supporting precision therapies and personalized mental health support. Crucially, integrating these findings into clinical diagnostic guidelines and algorithms for wearable technology and devices could significantly improve real-time, continuous monitoring of patient well-being.

## Data availability statement

The raw data supporting the conclusions of this article will be made available by the authors, without undue reservation.

## Ethics statement

The studies involving humans were approved by the Ethics Committee of the Institutional Review Board of the Institute of the Higher Nervous Activity and Neurophysiology of the Russian Academy of Sciences (protocol #1, 25 February 2021). The IHNA Ethics Committee was established on October 21, 2014, by the Order of the IHNA Director No. 77/a. The Committee ensures adherence to modern scientific ethics standards and includes leading scientists in various neurobiology fields https://www.ihna.ru/en/employees/employee-info/ethics. The studies were conducted in accordance with the local legislation and institutional requirements. The participants provided their written informed consent to participate in this study.

## Author contributions

EA: Data curation, Investigation, Visualization, Writing – original draft, Writing – review & editing. NZ: Formal analysis, Visualization, Writing – original draft, Writing – review & editing. IP: Funding acquisition, Writing – review & editing. OM: Conceptualization, Funding acquisition, Methodology, Project administration, Resources, Supervision, Writing – review & editing.
