# Supplementary material for: Heart rate response to cognitive load as a marker of depression and increased anxiety
Source: Front Psychiatry. 2024 Jul 11;15:1355846. doi: 10.3389/fpsyt.2024.1355846 (PMC11269089; doi:10.3389/fpsyt.2024.1355846)
Supplement: Supplementary file 1 [file DataSheet_1.pdf]

Supplementary Material

Heart rate response to cognitive load as a marker of depression and increased anxiety

Evgeniia I. Alshanskaia [1* †], Natalia A. Zhozhikashvili[2 †], Irina S. Polikanova[3], Olga V. Martynova[3,4,5]

[1]School of Psychology, Faculty of Social Sciences, HSE University, Moscow, Russia,
[2] Faculty of Social Sciences, Laboratory for Cognitive Research, HSE University, Moscow, Russia,
[3] Faculty of Biology and Biotechnology, HSE University, Moscow, Russia,
[4] Institute for Cognitive Neuroscience, HSE University, Moscow, Russia,
[5] Laboratory of Human Higher Nervous Activity, Institute of Higher Nervous Activity and Neurophysiology of the Russian Academy of Sciences, Moscow, Russia.

*** Correspondence:**
Evgenia I. Alshanskaia
eisokolova@hse.ru

Supplementary Data

Protocol

Research Date ____
Code _________________________ Date of birth___________________________
Height_________________________ Weight______________________________
Smoking ________________________Cycle day ___________________________
What time did you go to bed the night before the experiment? ______________________
What time did you wake up today____
Head injuries, sleep disorders, fainting, seizures, headaches__________________________
Are there any chronic diseases? ____
____
What medications are you currently taking? ___________________________________
Have you ever had anxiety attacks before? ____________________________________
Have you had episodes of depression before?__________________________________
Have you consulted a doctor/ or psychotherapist? ______________________________
Your education, profession ____

Time	Progress of the study	Notes
	Intro and time of the experiment	

	Informed consent and the completion of an anonymized protocol header	
	Epworth Sleepiness Scale	
	Beck scale	**Results**
	Clinical questionnaire	
	STAI	
	Connecting the equipment (ECG, PPG sensors) and explanation	
	**Background polygraph recording for two minutes**	
	Recording	
	Saving a record	
	**Block 1**	
	Recording	
	Saving a record	
	**Block 2**	
	Recording	
	Saving a record	
	Calculating results and winning amount money	
	Participant feedback and discussion	

Researchers signatures: ____

Notes:____

Impact of Gender Differences

Females (mean = 47.1, SD = 8.6) scored higher on the Spielberger Trait Anxiety Scale (W = 1349, p-value = 0.001218) than males (mean = 40.5, SD = 8).Females (mean = 10.8, SD = 7.9) scored higher on the Beck Depression Scale (U statistic = 1315.5, p-value = 0.01993) than males (mean = 6.6, SD = 4.1). We recalculated our models, adding the gender predictor to them. We retained significant results for the interaction of trait anxiety (STAI) and the average level of difficulty (3 colors), as well as the interaction of trait depression (BDI) and all levels of difficulty (2, 3, 4, 5, 6 colors, as compared to the easiest level), regardless of gender (ps < 0.05). We also retained significant results for the interaction of self-diagnosed depression episodes and task difficulty (2, 4, 6 colors), as well as psychiatrist-diagnosed depression episodes (6 colors, as compared to the easiest level), regardless of gender (ps < 0.05). However, results for the model with anxiety attacks were not replicated (interaction anxiety attacks * task difficulty: all ps > 0.05).
